# Presence of MDSC associates with impaired antigen-specific T cell reactivity following COVID-19 vaccination in cirrhotic patients

**DOI:** 10.3389/fimmu.2023.1287287

**Published:** 2023-10-20

**Authors:** Andreas Törnell, Elin Blick, Samer Al-Dury, Hanna Grauers Wiktorin, Johan Waern, Johan Ringlander, Sigrun Einarsdottir, Magnus Lindh, Kristoffer Hellstrand, Martin Lagging, Anna Martner

**Affiliations:** ^1^ Department of Microbiology and Immunology, Institute of Biomedicine, Sahlgrenska Academy, University of Gothenburg, Gothenburg, Sweden; ^2^ Department of Life Sciences, Chalmers University of Technology, Gothenburg, Sweden; ^3^ Department of Medicine, Gastroenterology and Hepatology Unit, Sahlgrenska University Hospital, Gothenburg, Sweden; ^4^ Department of Infectious Diseases, Institute of Biomedicine, Sahlgrenska Academy, University of Gothenburg, Gothenburg, Sweden; ^5^ Department of Clinical Microbiology, Region Västra Götaland, Sahlgrenska University Hospital, Gothenburg, Sweden; ^6^ Department of Hematology and Coagulation, Institute of Medicine, Sahlgrenska Academy, University of Gothenburg, Gothenburg, Sweden

**Keywords:** cirrhosis, MDSC, COVID-19, T cells, vaccination, immunosuppression

## Abstract

**Background and aims:**

Cirrhosis entails high risk of serious infections and abated efficiency of vaccination, but the underlying mechanisms are only partially understood. This study aimed at characterizing innate and adaptive immune functions, including antigen-specific T cell responses to COVID-19 vaccination, in patients with compensated and decompensated cirrhosis.

**Methods:**

Immune phenotype and function in peripheral blood from 42 cirrhotic patients and 44 age-matched healthy controls were analysed after two doses of the mRNA-based COVID-19 vaccines [BNT162b2 (Pfizer BioNTech) or mRNA-1273 (Moderna)].

**Results:**

Cirrhotic patients showed significantly reduced blood counts of antigen-presenting dendritic cells (DC) and high counts of monocytic myeloid-derived suppressor cells (M-MDSC) as compared to healthy controls. In addition, monocytic cells recovered from cirrhotic patients showed impaired expression of the antigen-presenting molecule HLA-DR and the co-stimulatory molecule CD86 upon Toll-like receptor (TLR) stimulation. These features were more prominent in patients with decompensated cirrhosis (Child-Pugh classes B & C). Interestingly, while patients with compensated cirrhosis (Child-Pugh class A) showed an inflammatory profile with myeloid cells producing the proinflammatory cytokines IL-6 and TNF, decompensated patients produced reduced levels of these cytokines. Cirrhotic patients, in particular those with more advanced end-stage liver disease, mounted reduced antigen-specific T cell reactivity to COVID-19 vaccination. Vaccine efficiency inversely correlated with levels of M-MDSC.

**Conclusion:**

These results implicate MDSC as mediators of immunosuppression, with ensuing deficiency of vaccine-specific T cell responses, in cirrhosis.

## 1 Introduction

Cirrhosis is associated with systemic inflammation and immunodeficiency, a state often referred to as cirrhosis-associated immune dysfunction (1, 2). This systemic inflammation is a consequence of persistent stimulation of pattern recognition receptors (PRR) of innate immune cells by damage-associated molecular patterns (DAMP) released from injured hepatocytes and pathogen-associated molecular patterns (PAMP) from translocating gut bacteria, resulting in enhanced production of proinflammatory cytokines (1, 3). Excessive stimulation of PRR, in particular repeated exposure to lipopolysaccharide (LPS), may eventually lead to a dampened responsiveness to further stimulation (4). The hallmarks of such LPS tolerance, which is commonly observed in advanced cirrhosis, include reduced ability of innate immune cells to produce proinflammatory cytokines upon re-exposure to LPS along with reduced expression of the antigen-presenting receptor HLA-DR (4–6). In turn, this may impair antigen presentation with consequently dampened adaptive immune responses (7). Studies on adaptive immune function in cirrhosis are limited, but recent reports describe T cell exhaustion and dysfunctional cytokine signalling (3, 4, 8). In addition to causing potentially inefficacious antigen presentation, chronic inflammation may also encompass abnormal myelopoiesis with ensuing expansion and activation of myeloid-derived suppressor cells (MDSC), i.e., immature myeloid cells that suppress T cell immunity (9). MDSC are known to have potent immunosuppressive activity associated with poor clinical outcomes in cancer (10) and COVID-19 (11). MDSC also have been suggested to play a role in the progression of liver disease (12, 13). Preliminary data indicate that higher levels correlate with more advanced liver fibrosis stage as well as inflammation grade (14).

During the initial phases of the SARS-CoV-2 pandemic and before widely available vaccination against COVID-19, patients with cirrhosis, particularly those with decompensated disease, were at significantly elevated risk for severe COVID-19 related morbidity and death (15). We and others have recently reported the reduced ability of cirrhotic patients to mount adaptive immune response following two doses of mRNA-based COVID-19 vaccination, but the underlying mechanisms remain largely unknown (16–18). This study determined the immune phenotype and function in peripheral blood in cirrhotic patients and healthy controls, collected after two doses of mRNA-based COVID-19 vaccination with the aim to identify factors of relevance to the poor vaccine response observed in this population. Our results implicate monocytic MDSC (M-MDSC) as mediators of reduced antigen-specific T cell response in cirrhosis.

## 2 Materials and methods

### 2.1 Study population

Sample collection was performed at Sahlgrenska University Hospital in Gothenburg, Sweden between March, and October 2021. Peripheral blood was collected from patients with cirrhosis (N=42) and age-matched healthy controls (N=44) after the second dose of mRNA-based COVID-19 vaccine (BNT162b2 [Pfizer BioNTech] or mRNA-1273 [Moderna]), and from unvaccinated healthy controls (N=11). Three healthy controls contributed samples before and after vaccination. Two patients with cirrhosis contracted COVID-19 infection, as reflected by seropositivity of IgG antibodies against the receptor-binding domain (RBD) within spike 1 (S1) (anti-RBD-S1 IgG), before vaccination and were excluded from analyses of S1-specific immunity. Remaining patients and controls were infection naïve.

Patients with cirrhosis were stratified using Child-Pugh Score (CPS) (19). In this study, we define patients with CPS A (5 – 6 points) as patients with compensated cirrhosis, while patients with CPS B (7 – 9 points) or CPS C (10 – 12 points) that had previously encountered one or several liver-related complications, as patients with decompensated cirrhosis. Characteristics of study participants, including age, sex, MELD score, disease etiology and use of immunosuppressive drugs, are detailed in **Table 1**. All participants gave written informed consent before enrolment and the study was performed in accordance with the declaration of Helsinki. Study approval was obtained from the Swedish Ethical Review Authority (Etikprövningsmyndigheten; permit number 2021-00539), from the Swedish Medical Products Agency (Dnr: 5.1-2021-11118 and EudraCT no. 2021-000349-42) and is registered at the European Union Drug Regulating Authorities Clinical Trials Database (EudraCT no. 2021-000349-42).

**Table 1:** Characteristics of included participants.

	Patients with Cirrhosis	Control Cohort
	n=42	n=44
**Age, median (range)**	63 (25-75)	59 (25-86)
**Females, n (%)**	22 (52)	28 (64)
**Pfizer vaccine ^a^ , n (%)**	39 (93)	39 (89)^b^
**Days from second vaccine dose to sampling, median (range)**	88 (14-109)	28 (14-147)
**Child-Pugh class A, n (%)**	28 (67)	
**Child-Pugh class B, n (%)**	11 (26)	
**Child-Pugh class C, n (%)**	3 (7)	
**MELD ^c^ score, median (range)**	8 (6-17)	
**Immunosuppressive drugs, n (%)**	5 (12)	
Etiology, n (%)		
** Alcohol**	24 (57)	
**NASH ^d^**	5 (12)	
** Alcohol+NASH**	3 (7)	
** Cholestatic liver disease**	4 (10)	
** Autoimmune hepatitis**	2 (5)	
** Hepatitis C**	2 (5)	
** Cryptogenic cirrhosis**	2 (5)	

a BNT162b2 (Pfizer BioNTech).

b One individual was given ChAdOx1 (AstraZeneca) and mRNA-1273 (Moderna) vaccine as first and second doses, respectively.

c Model for end stage liver disease.

d Nonalcoholic steatohepatitis.

### 2.2 Leukocyte isolation

Peripheral blood mononuclear cells (PBMC) were isolated from blood samples collected in BD Vacutainer lithium-heparin tubes (BD) from 30 cirrhotic patients and 25 healthy controls or from 5 healthy blood donor buffy coats acquired from Sahlgrenska Blood Centre (Gothenburg, Sweden). Samples were diluted 1:1 in buffered sodium chloride and layered on Lymphoprep (STEMCELL Technologies) for density gradient centrifugation. Buffy coat samples were separated with dextran sedimentation prior to density gradient centrifugation. PBMC were then recovered and used immediately or cryopreserved in Recovery Cell Culture freezing medium (Life Technologies) and stored at -140°C until further analysis.

T cells were purified from healthy blood donor buffy coat PBMC using an anti-CD3 magnetic particle separation kit (Miltenyi Biotec), according to manufacturer’s instructions. Monocytes from cirrhotic patients and healthy controls were purified from cryo-recovered PBMC using a pan monocyte isolation kit (Miltenyi Biotec) according to the manufacturer’s instructions with the following modifications. After biotin incubation, buffer volume added was reduced to 20 μL/10 million cells and magnetic bead volume increased to 30 μL/10 million cells. T cell purity (CD3-positive cells) exceeded 99% and monocyte purity (CD14-positive cells) exceeded 78%.

### 2.3 PBMC stimulation and immunophenotyping by flow cytometry

Cryo-recovered PBMC were cultured at 2.5×10^6^ cells/mL in presence of 0.1 μg/mL LPS (eBioscience), 1 μg/mL of the synthetic toll-like receptor agonist CL097 (*In vivo*gen), or no stimuli in round-bottom 96-well microplates at 37°C and 5% CO_2_ for five hours. Two patients with cirrhosis with insufficient cell numbers after cryo-recovery were not stimulated with LPS. After stimulation, supernatants were collected and cells were stained with a panel of antibodies comprising anti-CD1c (clone L161; BioLegend), anti-CD11b-PE (clone ICRF44; BD Biosciences), anti-CD16-BV605 (clone 3G8; BD Biosciences), anti-CD14-APC-Cy7 (clone MφP-9; BD Biosciences), anti-CD141-APC (clone 1A4; BD Biosciences), anti-CD33-PE-Cy7 (clone P67.6; BD Biosciences), anti-CD86-BV711 (clone 2331; BD Biosciences), anti-HLA-DR-BV786 (clone G46-6; BD Biosciences), anti-PD-L1-BUV395 (clone MIHI; BD Biosciences), anti-CD19-PerCP-Cy5.5 (clone SJ25C1; BD Biosciences), and Live/Dead™ Yellow (Life Technologies). Flow cytometric analysis was performed on a five-laser BD LSRFortessa (BD Biosciences). Gatings of cell populations were set based on an internal PBMC control that was run in each experiment.

### 2.4 IL-6 and TNF enzyme-linked immunosorbent assay

Levels of interleukin-6 (IL-6) and tumour necrosis factor (TNF) in supernatants from unstimulated or LPS- or CL097-stimulated PBMC were determined by enzyme-linked immunosorbent assays (ELISA) (R&D systems) according to the manufacturer’s instructions. Optical density was measured using a FLUOstar Omega Microplate Reader (BMG).

### 2.5 Multiplex cytokine analysis in whole blood

Whole blood samples from unvaccinated healthy controls as well as from patients with cirrhosis and healthy controls after two doses of COVID-19 mRNA vaccine were stimulated with 1 μg/mL/peptide of 15-mer peptides with 11-amino acid overlap spanning the N-terminal S1 domain of the SARS-CoV-2 surface glycoprotein (S1-peptides; 130-127-041, Miltenyi Biotec), 1μg/mL/peptide of 15-mer peptides with 11-amino acid overlap spanning the complete sequence of the pp65 protein of human cytomegalovirus (CMV-peptides; 130-093-435, Miltenyi Biotec), or no stimuli, as described previously (20). Following incubation, samples were centrifuged at 1,500rpm for five minutes and plasma was collected and stored at -80°C until further analyses. Levels of interferon-γ (IFN-γ), IL-2, IL-5 and IL-9 in plasma samples were measured by FirePlex (ab243549 and ab285173, Abcam) on a five-laser BD LSRFortessa (BD Biosciences), according to the manufacturer’s instructions. Haemolysis occurred in samples from two healthy controls, these samples were therefore not analysed for cytokine content.

### 2.6 T cell apoptosis assay

T cells from healthy blood donors were co-cultured at 0.25×10^6^ cells/mL with monocytes from a subset of cirrhotic patients (N=9) or healthy controls (N=9) in round-bottom ultra-low attachment 96-well microplates (7007, Corning). Cells were incubated overnight at 37°C and 5% CO_2_, and subsequently stained with an antibody panel comprising Live/Dead™ Yellow (Life Technologies), anti-CD14-PE-Cy7 (clone M5E2; BD Biosciences) or anti-CD14-FITC (clone: MφP9; BD Biosciences) and anti-CD3-FITC (clone: HIT3a; BD Biosciences) or anti-CD3-BUV496 (clone: UCHT1; BD Biosciences), and analysed on a five-laser BD LSRFortessa (BD Biosciences).

### 2.7 Serology

Serum samples were collected from patients and healthy controls in conjunction with samples for whole blood stimulation and PBMC isolation. Anti-CMV IgG and SARS-CoV-2 anti-RBD-S1 IgG in sera were quantified using the Alinity i instrument (Abbott, Abbott Park, Il, USA), with the CMV IgG and SARS-CoV-2 IgG II Quant kits, respectively, and reported as concentrations of arbitrary units (AU/mL) for CMV IgG and binding antibody units (BAU/mL) for SARS-CoV-2 RBD-S1 IgG. For one patient, no serum sample was available for quantification of SARS-COV-2 IgG. CMV IgG was quantified from a subset of healthy controls (N=25) and cirrhotic patients (N=31).

### 2.8 Data analysis

Statistical analyses were performed using GraphPad Prism® version 8 or IBM SPSS statistics 28.0. Flow cytometric data were analysed with FlowJo software, version 10 or later (BD Biosciences). Kruskal-Wallis’ test with Dunn’s multiple comparisons test was used for comparisons between multiple groups. To analyse effects of potential confounders on S1-specific T cell reactivity, the impact of age, sex, and time from the second vaccine dose to sampling was determined using linear regression. Variables with a P-value <0.1 were included in a multiple linear regression model with disease status. In this multivariable analysis, patients were analysed as one group, and logarithmic values of S1-induced cytokines were used. A linear mixed-effects model analysed the difference of T cell viability between patients with cirrhosis and healthy controls. Subject and T cell donor were included as random effects while cell ratio and disease status were included as fixed effects. The association between continuous parameters was determined using Spearman’s rank correlation. All stimuli-induced and antigen-induced immune responses are presented as stimuli/antigen-specific, with levels in unstimulated samples subtracted. Data are presented as mean ± SEM. P-values were designated as follows: *P<0.05, **P<0.01, ***P<0.001, ****P<0.0001.

## 3 Results

### 3.1 Immunophenotyping reveals accumulation of M-MDSC and reduction of dendritic cells in patients with cirrhosis

**Figure 1:**
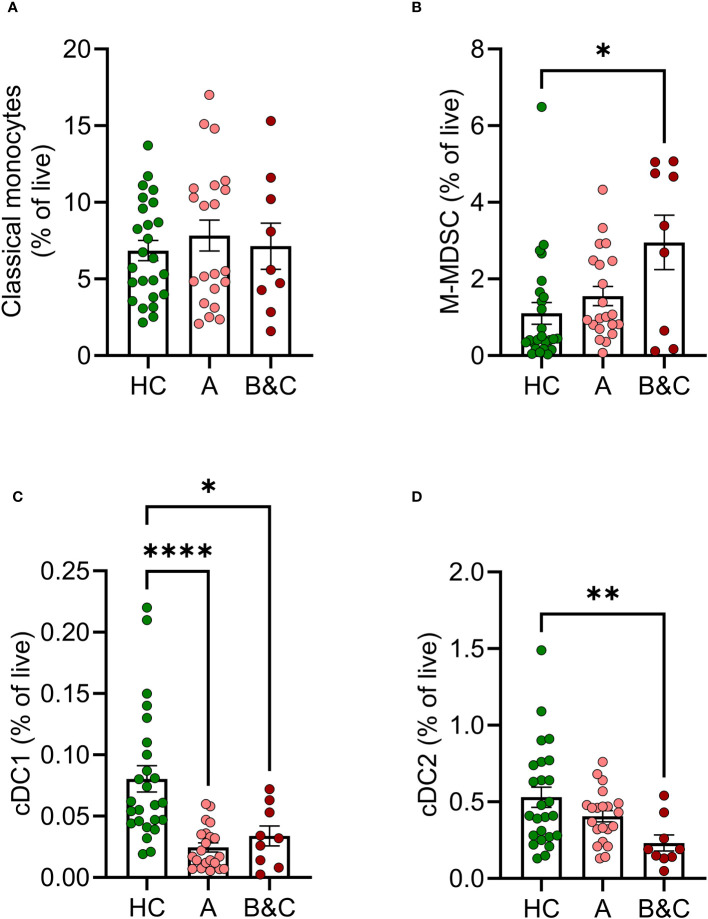
Frequencies of innate immune cells are altered in cirrhosis. Frequencies of **(A)** classical monocytes, **(B)** M-MDSC, **(C)** cDC1 and **(D)** cDC2s in peripheral blood mononuclear cells from healthy controls (HC, N=25) and patients with cirrhosis (N=30) were determined by flow cytometry after five hours of culture. Cirrhotic patients are grouped by Child-Pugh class with patients in class A in one group (A, N=21), and class B and C in one group (B&C, N=9). Statistical analyses were performed using Kruskal-Wallis’ test with Dunn’s multiple comparisons test. *P<0.05, **P<0.01, ****P<0.0001.

The phenotype and function of myeloid cell populations in peripheral blood of cirrhotic patients were compared with myeloid cells in age-matched healthy donors. While the levels of classical monocytes (CD33^+^ CD14^+^ CD11b^+^ CD16^-^) were not altered, patients with cirrhosis, in particular those with decompensated disease, showed increased frequency of M-MDSC (CD33^+^CD14^+^CD11b^+^HLA-DR^low^) along with reduced frequencies of both conventional dendritic cells type 1 (cDC1) (CD33^+^ CD14^-^ HLA-DR^+^ CD141^+^ CD1c^-^) and type 2 (cDC2) (CD33^+^ CD14^-^ HLA-DR^+^ CD141^-^ CD1c^+^) (**Figure 1**). The gating strategies for classical monocytes, M-MDSC and dendritic cells are shown in **Supplementary Figure 1**. Patients with compensated cirrhosis showed trends towards similar alterations in myeloid cell populations and harboured significantly fewer cDC1 than healthy controls (**Figure 1**).

**Figure 2:**
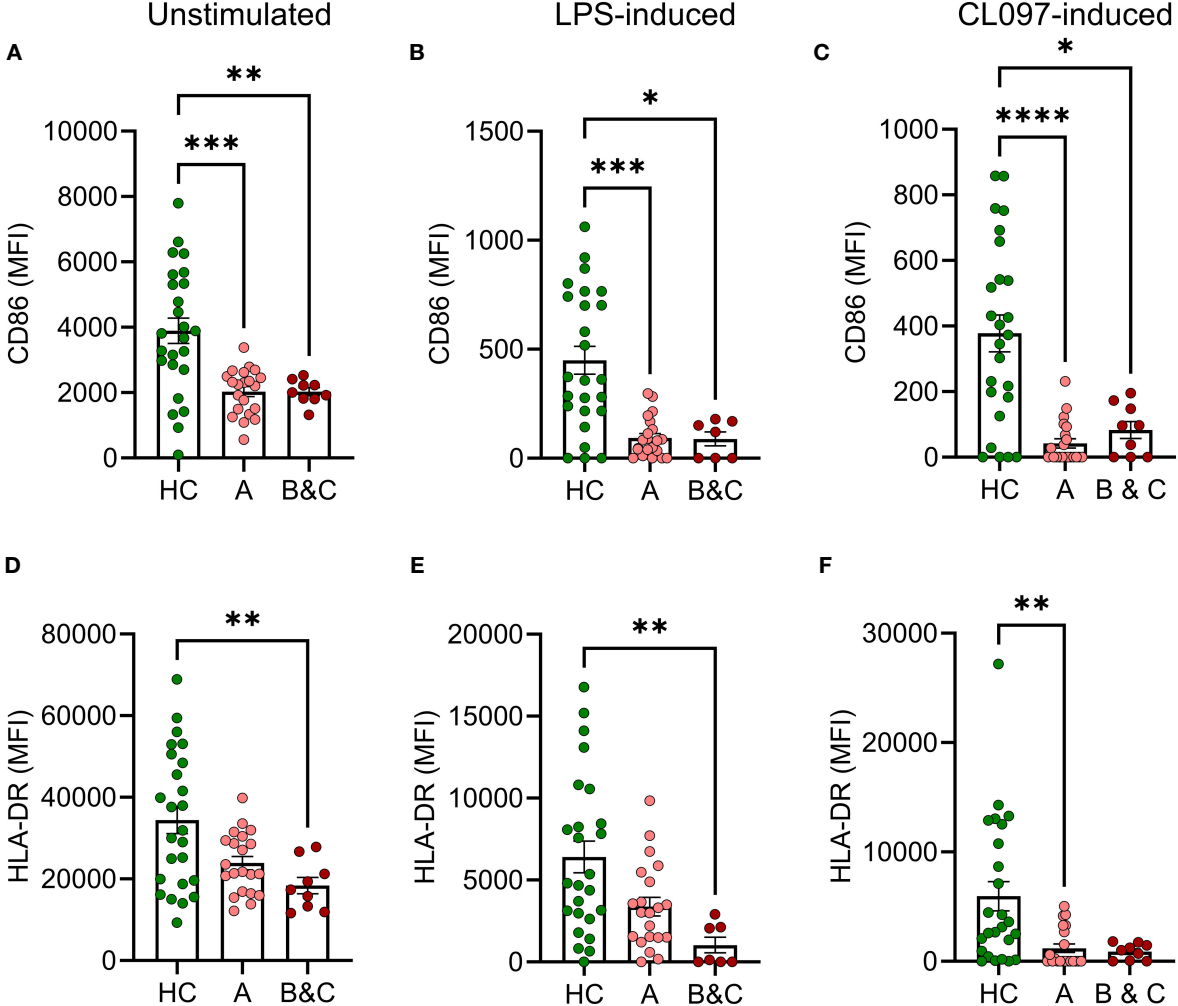
Reduced basal and LPS-induced expression of CD86 and HLA-DR on monocytes from cirrhotic patients. **(A, D)** Basal, **(B, E)** LPS-induced or **(C, F)** CL097-induced expression of **(A–C)** CD86 or **(D–F)** HLA-DR on classical monocytes from healthy controls (HC, N=25) and patients with cirrhosis (N=30) were determined by flow cytometry after five hours of culture. Cirrhotic patients are grouped by Child-Pugh class with patients in class A in one group (A, N=21), and class B and C in one group (B&C, N=9). Statistical analyses were performed using Kruskal-Wallis’ test with Dunn’s multiple comparisons test. *P<0.05, **P<0.01, ***P<0.001, ****P<0.0001. MFI, median fluorescence intensity.

Monocytic cells from patients with cirrhosis showed reduced basal expression of CD86 and impaired ability to mount PAMP-induced CD86 compared with healthy controls at all stages of disease (**Figures 2A–C**). The PAMPs utilized were the bacterial TLR-4 agonist LPS and CL097 which binds TLR-7/8 that recognize viral single stranded RNA (21). Similarly, the basal and induced expression of HLA-DR was reduced or tended to be reduced in patients with cirrhosis (**Figures 2D–F**). Activation marker expression on cDC2 in patients with cirrhosis and healthy donors did not differ significantly at baseline, but upon stimulation with CL097, cDC2 were less prone to upregulate expression of CD86 and HLA-DR (**Supplementary Figure 2**).

### 3.2 Inflammatory cytokine formation in early-stage cirrhosis

**Figure 3:**
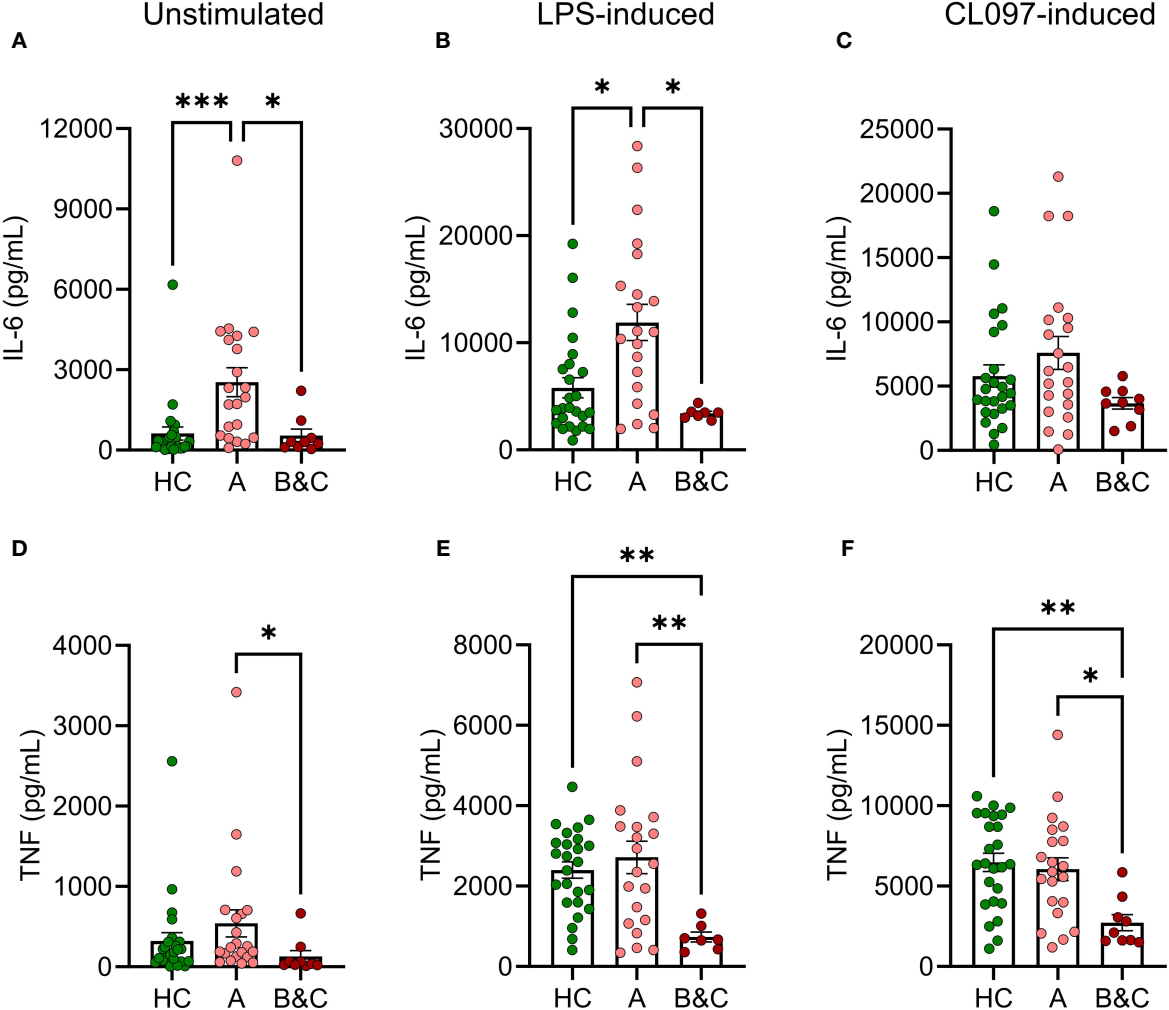
Basal and LPS-induced levels of IL-6 and TNF are increased in early-stage cirrhosis. PBMC from healthy controls (HC, N=25) and cirrhotic patients (N=30) were cultured for five hours after which **(A, D)** basal, **(B, E)** LPS-induced or **(C, F)** CL097-induced expression of **(A–C)** of IL-6 and **(D–F)** TNF were measured with ELISA. Cirrhotic patients are grouped by Child-Pugh class with patients in class A in one group (A, N=21), and class B and C in one group (B&C, N=9). Statistical analyses were performed using Kruskal-Wallis’ test with Dunn’s multiple comparisons test. *P<0.05, **P<0.01, ***P<0.001.

Levels of IL-6 and TNF were measured in PBMC culture supernatants after five hours of PAMP stimulation. Patients with compensated cirrhosis demonstrated increased basal and LPS-induced production of IL-6 compared to healthy controls and patients with more advanced disease (Child-Pugh class B & C) (**Figures 3A, B**). Similarly, the basal and LPS-induced production of TNF was reduced in patients with advanced cirrhosis (**Figures 3D, E**). Similar results, with trends towards enhanced production of proinflammatory cytokines in patients with compensated cirrhosis, and reduced production in patients with decompensated disease were obtained upon CL097 stimulation (**Figures 3C, F**).

### 3.3 Deficient T cell reactivity in patients with cirrhosis following COVID-19 mRNA vaccination

**Figure 4:**
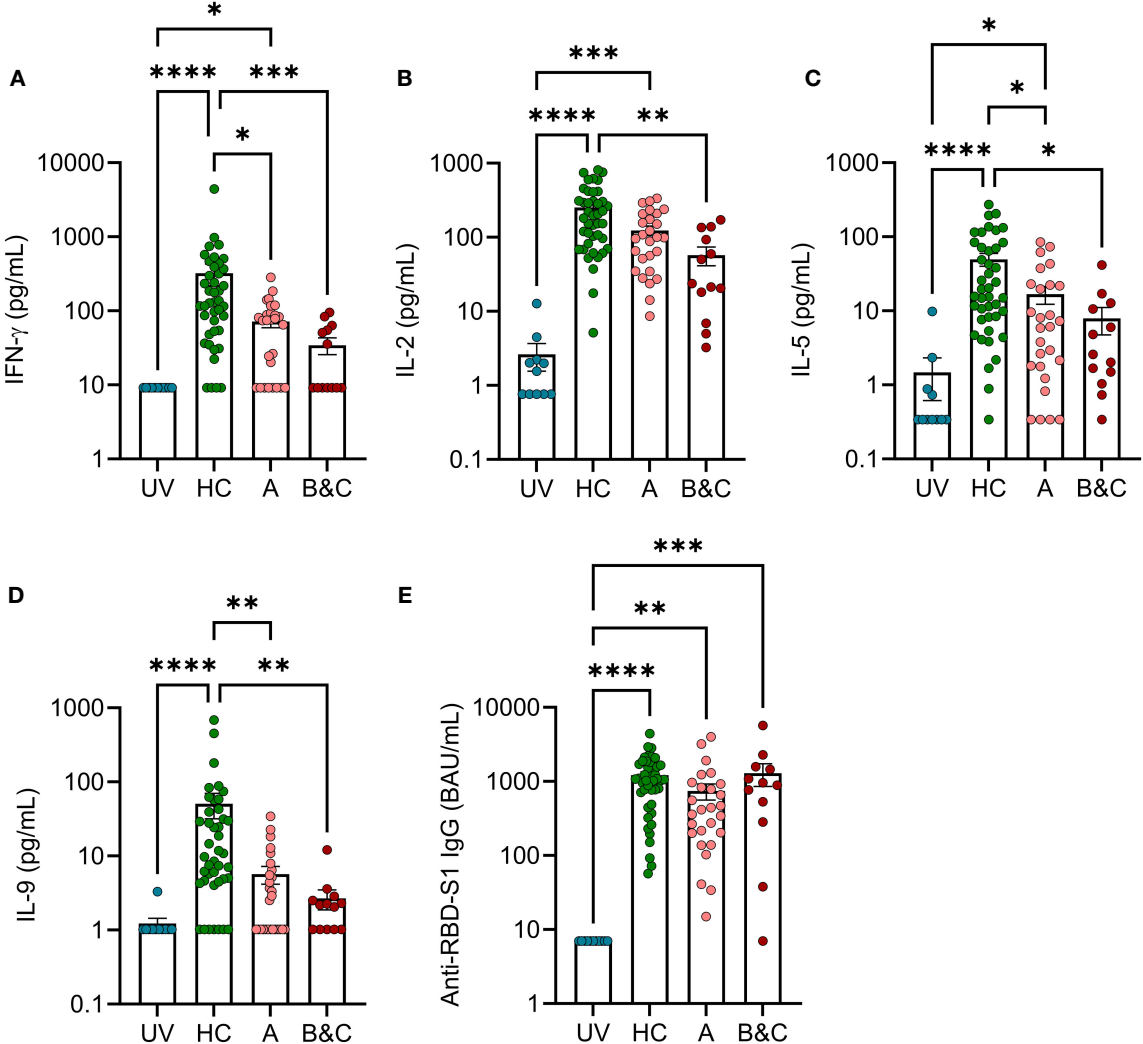
Two doses of COVID-19 vaccine fail to trigger robust T cell reactivity in response to S1-peptide stimulation in patients with advanced cirrhosis. SARS-COV-2 S1 peptide-induced formation of **(A)** IFN-γ **(B)** IL-2, **(C)** IL-5 and **(D)** IL-9 in unvaccinated (UV, N=11) or vaccinated healthy controls (HC, N=42), and cirrhotic patients (N=40) were determined by FirePlex. **(E)** Levels of anti-RBD S1 IgG in unvaccinated (UV, N=11) or vaccinated healthy controls (HC, N=44), and cirrhotic patients (N=39). Cirrhotic patients are grouped by Child-Pugh class with patients in class A in one group (A, N=27), and class B and C in one group (B&C, N=13 [cytokines] or N=12 [anti-RBD-S1 IgG]). Statistical analyses were performed using Kruskal-Wallis’ test with Dunn’s multiple comparisons test. *P<0.05, **P<0.01, ***P<0.001, ****P<0.0001.

To determine the presence of COVID-19 mRNA vaccine-induced antigen-specific T cells in the blood of patients with cirrhosis, a whole blood cytokine release assay was utilized (20, 22). After stimulation with multimeric peptides spanning the S1 region of SARS-CoV-2, vaccinated cirrhotic patients produced significantly lower levels of the S1-induced T cell cytokines IFN-γ, IL-2, IL-5, and IL-9, compared with vaccinated healthy controls. The deficiency of antigen-specific T cell reactivity was present in compensated cirrhosis and was more pronounced in patients with severe disease (**Figures 4A–D**). T cell reactivity among patients with cirrhosis remained significantly lower than healthy controls when accounting for potential confounders including age, sex, and time from the second vaccine dose to sampling. As previously reported (18), the S1-specific antibody response (anti-RBD-S1 IgG) after two doses of COVID-19 mRNA vaccine was not reduced in cirrhotic patients and, in contrast to the T cell responses, patients with severe disease did not show reduced ability to mount anti-RBD-S1 IgG (**Figure 4E**).

### 3.4 Presence of immunosuppressive M-MDSC correlates with poor COVID-19 vaccine-induced T cell responses

**Figure 5:**
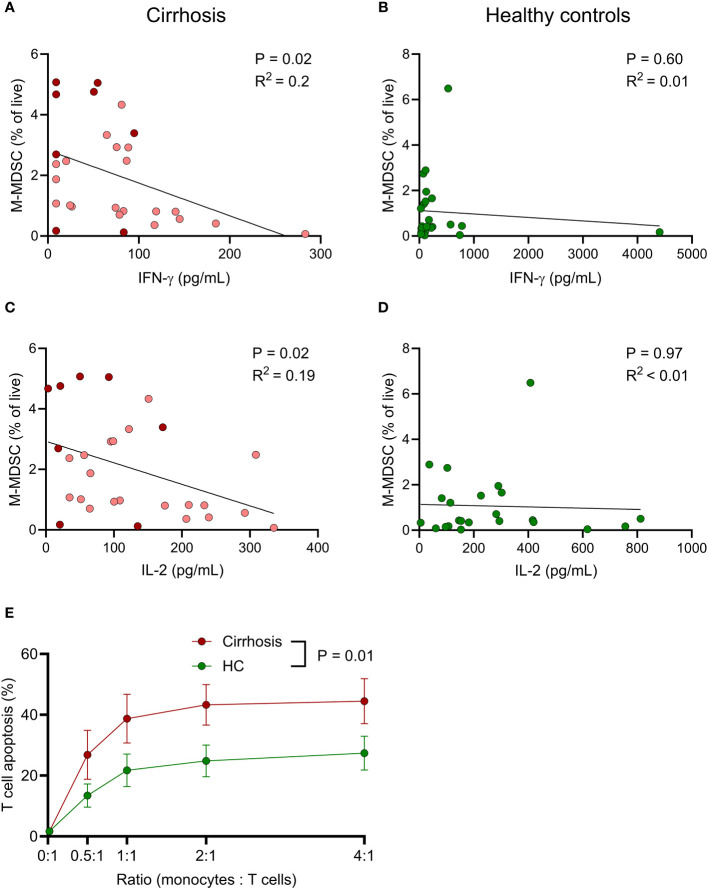
M-MDSC from cirrhotic patients potently induce T cell apoptosis and frequency in cirrhotic patients negatively correlate with vaccine-induced T cell immune responses. Associations between frequency of M-MDSC and spike 1 peptide-induced **(A, B)** IFN-γ and **(C, D)** IL-2 in **(A, C)** cirrhotic patients (N=28) and **(B, D)** healthy controls (N=23). **(A, C)** Light red; Child-Pugh class A (N=20), dark red; Child-Pugh class B&C (N=8). **(E)** T cells from healthy blood donors (N=5) were co-cultured with monocytes from cirrhotic patients (N=9) or healthy controls (HC, N=9) at different ratios for 24 h, after which T cell apoptosis was determined by flow cytometry. Statistics by **(A–D)** Spearman’s rank correlation or **(E)** linear mixed effects model.

Possible association between innate immunity and COVID-19 vaccine-induced T cell reactivity was analysed using Spearman’s rank correlation. Higher frequencies of M-MDSC in patients with cirrhosis correlated with reduced levels of S1-induced IFN-γ and IL-2 (**Figures 5A, C**). A similar albeit non-significant trend was seen for IL-5. Such correlations were not observed among healthy controls (**Figures 5B, D**), suggesting that M-MDSC in cirrhosis were more T cell-suppressive compared to those of healthy donors. No correlations between anti-RBD-S1 IgG and frequency of M-MDSC were observed in patients or controls (P >0.5 for both).

To compare M-MDSC-induced immunosuppression, monocytes were isolated from patients with cirrhosis and healthy controls and co-cultured with T cells at different ratios overnight, followed by assessment of T cell viability. Monocytes obtained from patients with cirrhosis were significantly more suppressive towards T cells than monocytes from healthy donors (**Figure 5E**).

To elucidate whether T cell immunity acquired prior to the development of cirrhosis was impaired in patients, whole blood samples from patients with cirrhosis and healthy controls with or without confirmed previous cytomegalovirus (CMV) infection, as defined by presence or absence of anti-CMV IgG antibodies in serum, were stimulated with CMV peptides. Samples from seronegative cirrhotic patients (N=7/31, 23%) and healthy controls (N=5/25, 20%) were pooled as a single control group. Induction of IFN-γ and IL-2 was seen in seropositive but not seronegative individuals (**Supplementary Figures 3A, B**). There was no significant difference in levels of CMV-induced IFN-γ or IL-2, or serum anti-CMV IgG between seropositive cirrhotic patients and healthy controls, although patients with advanced disease (CPS B & C) tended to produce lower levels of CMV-induced IL-2 (**Supplementary Figures 3A–C**). No correlations between frequency of M-MDSC and CMV peptide-induced production of IFN-γ or IL-2 in cirrhotic patients or healthy controls were observed (**Supplementary Figures 3D–G**).

## 4 Discussion

During the COVID-19 pandemic, it became evident that patients with liver cirrhosis experienced more severe respiratory disease after infection with SARS-CoV-2 compared with other groups. Hence, in a European registry study the case fatality rate for unvaccinated cirrhotic patients was 31%, with enhanced risk for patients above 65 years (44%) and those presenting with severe disease (Child-Pugh class C, 57%) (23). Postvaccination, the COVID-19 mortality in cirrhotic patients has clearly decreased, but still many patients require hospitalization following COVID-19 and the case fatality rate remains higher than for the general population (24). The incomplete protection may be due to a partly impaired ability to mount adaptive immune responses following COVID-19 mRNA vaccination (15–18). Cirrhotic patients are also less prone to mount protective immune responses following other vaccinations, including hepatitis B, pneumococcal and influenza vaccine (25–27). Booster doses are likely of clinical value, since upon repeated rounds of vaccination, there seems to be a catch-up effect of antibody levels in cirrhotic patients (27, 28). Whether a similar catch-up effect occurs also for cellular immune responses is less studied, but we and others have noted that the cellular immune responses are more impaired than the humoral immune responses in vaccinated cirrhotic patients (17, 18). The hallmarks of immune dysfunction in cirrhosis comprise systemic inflammation and immune exhaustion, but the mechanisms that translate systemic inflammation into deficient adaptive immunity are only partly understood (1). Here, we studied innate and adaptive immune profiles in peripheral blood cells from cirrhotic patients and healthy controls collected after COVID-19 mRNA vaccination to identify factors contributing to the dysfunctional T cell response characteristic of cirrhosis.

Functional antigen presentation is required to mount adaptive immunity following vaccination. In accordance with previous results, we observed reduced frequencies of dendritic cells (DC) in cirrhotic patients (6). The reduction was more pronounced in patients with decompensated cirrhosis (Child-Pugh class B&C) where frequencies of cDC1 and cDC2 were significantly lower than in healthy controls. Furthermore, classical monocytes from cirrhotic patients showed reduced basal and antigen-induced expression of HLA-DR and CD86 with similar albeit non-significant trends for cDC2. The diminished HLA-DR expression and expression of co-stimulatory molecules on antigen-presenting cells are likely to contribute to deficient priming of T cells (7, 29).

Cirrhosis is assumed to entail systemic long-term activation of innate immune cells through DAMP and PAMP stemming from damaged liver cells and from intestinal barrier dysfunction with ensuing systemic inflammation (1). This may in turn lead to expansion of MDSC with impaired phagocytic function along with T cell-suppressive features (30). We found that cirrhotic patients had increased blood levels of M-MDSC, in particular in more advanced end-stage liver disease. Similar findings were reported in a recent study where M-MDSC were found to associate with progression of liver failure (13). Furthermore monocytes, that include M-MDSC, from cirrhotic patients were significantly more T cell-suppressive than those isolated from healthy controls. As M-MDSC may exert immunosuppression toward T cells through release of reactive oxygen species and other immunosuppressive mediators (9, 30, 31), our results may translate into the identification of strategies to reduce M-MDSC counts and/or their mediators to ameliorate immune dysfunction in cirrhosis. This may be particularly relevant in the context of vaccination of patients with decompensated cirrhosis to improve vaccine efficacy.

Previous studies of proinflammatory cytokines in cirrhotic patients have yielded inconsistent results, particularly concerning cytokine levels in serum and levels produced in PBMC cultures following antigen stimulation *ex vivo* (32–34). In this study, we noted increased basal and LPS-induced proinflammatory cytokine signalling in compensated (Child-Pugh class A) but not decompensated (Child-Pugh class B&C) cirrhosis. A dysfunctional innate immune activation in Child-Pugh class B&C patients was observed in our study as reflected by lack of TLR-induced IL-6 and TNF production. These findings are in line with a dynamic profile of cirrhosis comprising systemic inflammation in early stages followed by immune exhaustion as evidenced by, for example, LPS tolerance in later stages (1, 4).

COVID-19 mRNA vaccine-induced antigen-specific T cell reactivity in cirrhotic patients and healthy controls was measured using a cytokine release assay wherein whole blood samples were stimulated *ex vivo* with SARS-CoV-2 S1 peptides. The subsequent cytokine formation was measured in supernatant plasma. In this assay, IL-2 is almost exclusively contributed by CD4^+^ T cells (35). Additionally, S1-induced IFN-γ, IL-5 and IL-9 likely capture adaptive T cell reactivity as evidenced by S1 induction in vaccinated but not in unvaccinated individuals. In accordance with previous studies of cirrhotic patients, in particular those with decompensated disease exhibited reduced COVID-19 mRNA vaccine-induced T cell reactivity compared with healthy controls (17, 18). Due to logistical difficulties stemming from the regional vaccination strategy for risk groups, the time from COVID-19 vaccination to sample collection varied among cirrhotic patients and healthy controls. The difference in T cell reactivity remained significant when adjusting for potential confounders including time from the second vaccine dose to sampling. The vaccine-induced levels of anti-RBD-S1 IgG were less affected in cirrhotic patients and the ability to mount S1-specific antibodies did not differ based on severity of cirrhosis. These results thus suggest that more advanced end-stage liver disease (Child-Pugh class B&C) associates with inability to mount vaccine-specific T cell responses. A key finding was that the frequency of M-MDSC significantly correlated with S1-induced IFN-γ and IL-2 in cirrhotic patients, but not in healthy controls, which is in line with a more immunosuppressive MDSC phenotype in cirrhosis. This notion was corroborated by the finding that monocytes from patients with cirrhosis more potently induced apoptosis in healthy heterologous T cells compared with monocytes from healthy controls. Notably, levels of M-MDSC in cirrhotic patients did not correlate with levels of anti-RBD S1 IgG, suggesting that M-MDSC mainly suppress acquired cellular but not humoral immune responses. This conclusion is supported by the finding that twice-vaccinated cirrhotic patients mainly showed an impaired COVID-19 specific T cell response but an intact antibody response.

To determine if T cell reactivity towards antigens encountered prior to cirrhosis development might be impaired in cirrhotic patients, T cell responses to CMV peptides were measured in patients that had undergone CMV infection, where the primary infection typically occurs in childhood or adolescence (36). T cell responses to CMV were not defect in cirrhotic patients and did not correlate to the frequency of M-MDSC. We thus speculate that expansion of M-MDSC and related immune dysfunction mainly affects the initiation of adaptive immunity while previously developed adaptive immune response may remain intact.

By analysing the innate cell immunophenotype and function in conjunction with data on COVID-19 mRNA vaccine-induced adaptive reactivity, we were able to unravel integrated aspects of immune dysfunctions in patients with cirrhosis. Limitations to the study include the variable time from second vaccine dose to sampling, that the number of study participants is relatively low, and that PBMC were not available from the entire cohort for immunophenotyping. Despite these reservations, our results may point towards the identification of potentially targetable mechanisms of immune dysfunction in cirrhosis.

## Data availability statement

The raw data supporting the conclusions of this article will be made available by the authors, without undue reservation.

## Ethics statement

The studies involving humans were approved by the Swedish Ethical Review Authority (Etikprövningsmyndigheten; permit number 2021-00539), from the Swedish Medical Products Agency (Dnr: 5.1-2021-11118 and EudraCT no. 2021-000349-42) and is registered at the European Union Drug Regulating Authorities Clinical Trials Database (EudraCT no. 2021-000349-42). The studies were conducted in accordance with the local legislation and institutional requirements. The participants provided their written informed consent to participate in this study.

## Author contributions

AT: Data curation, Formal Analysis, Investigation, Methodology, Supervision, Visualization, Writing – original draft, Writing – review & editing. EB: Data curation, Formal Analysis, Investigation, Methodology, Visualization, Writing – original draft, Writing – review & editing. SA-D: Conceptualization, Data curation, Investigation, Project administration, Writing – review & editing. HW: Investigation, Methodology, Supervision, Writing – review & editing. JW: Conceptualization, Data curation, Investigation, Project administration, Writing – review & editing. JR: Funding acquisition, Investigation, Writing – review & editing. SE: Conceptualization, Data curation, Investigation, Project administration, Writing – review & editing. MLi: Conceptualization, Supervision, Writing – review & editing. KH: Conceptualization, Supervision, Writing – original draft, Writing – review & editing. MLa: Conceptualization, Funding acquisition, Supervision, Writing – review & editing. AM: Conceptualization, Formal Analysis, Funding acquisition, Project administration, Supervision, Writing – original draft, Writing – review & editing.
