# Supplementary material for: Presence of MDSC associates with impaired antigen-specific T cell reactivity following COVID-19 vaccination in cirrhotic patients
Source: Front Immunol. 2023 Oct 20;14:1287287. doi: 10.3389/fimmu.2023.1287287 (PMC10623131; doi:10.3389/fimmu.2023.1287287)
Supplement: Supplementary file 1 [file DataSheet_1.docx]

Supplementary Material

## Supplementary Figures

**
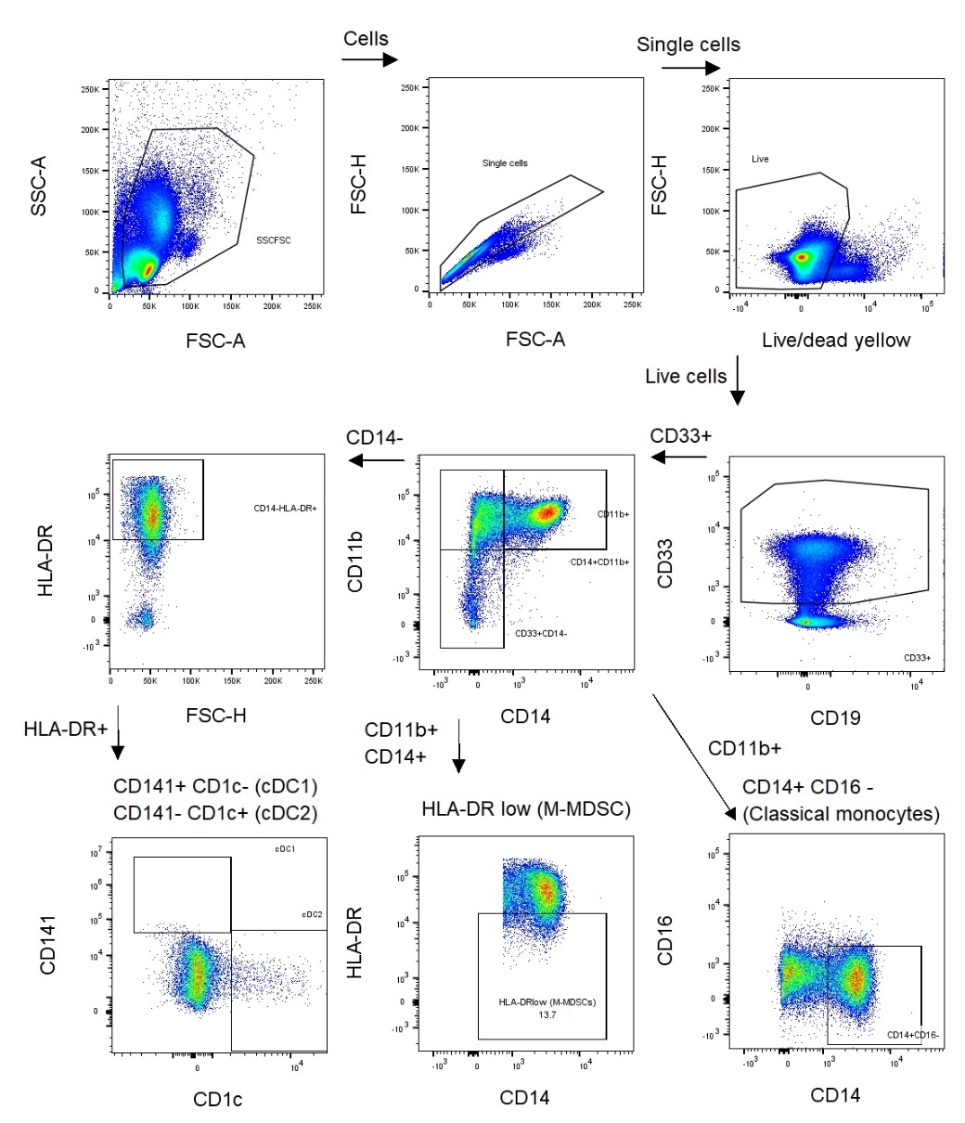
A**


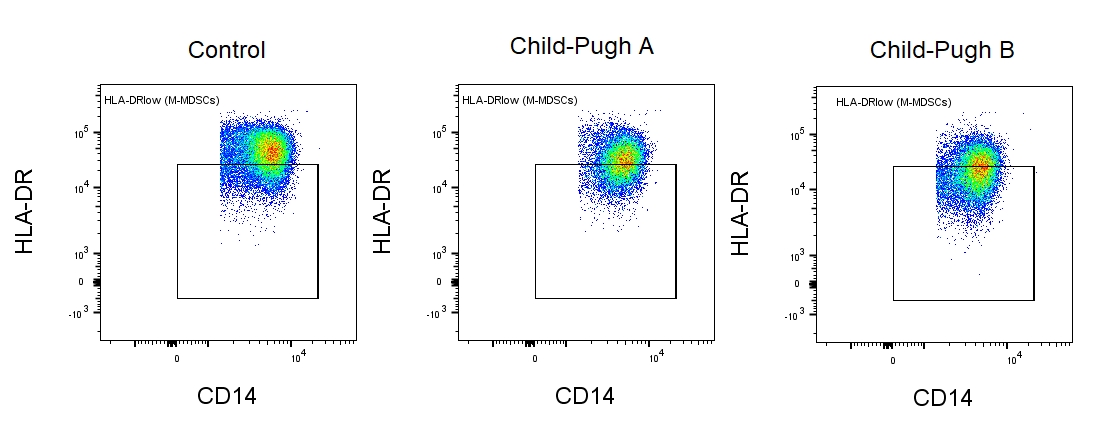


**B**

**Supplementary figure 1. A**, Gating strategy for classical monocytes, cDC1, cDC2 and M-MDSC. **B**, Representative M-MDSC gatings in a healthy control, Child-Pugh class A and class B sample.


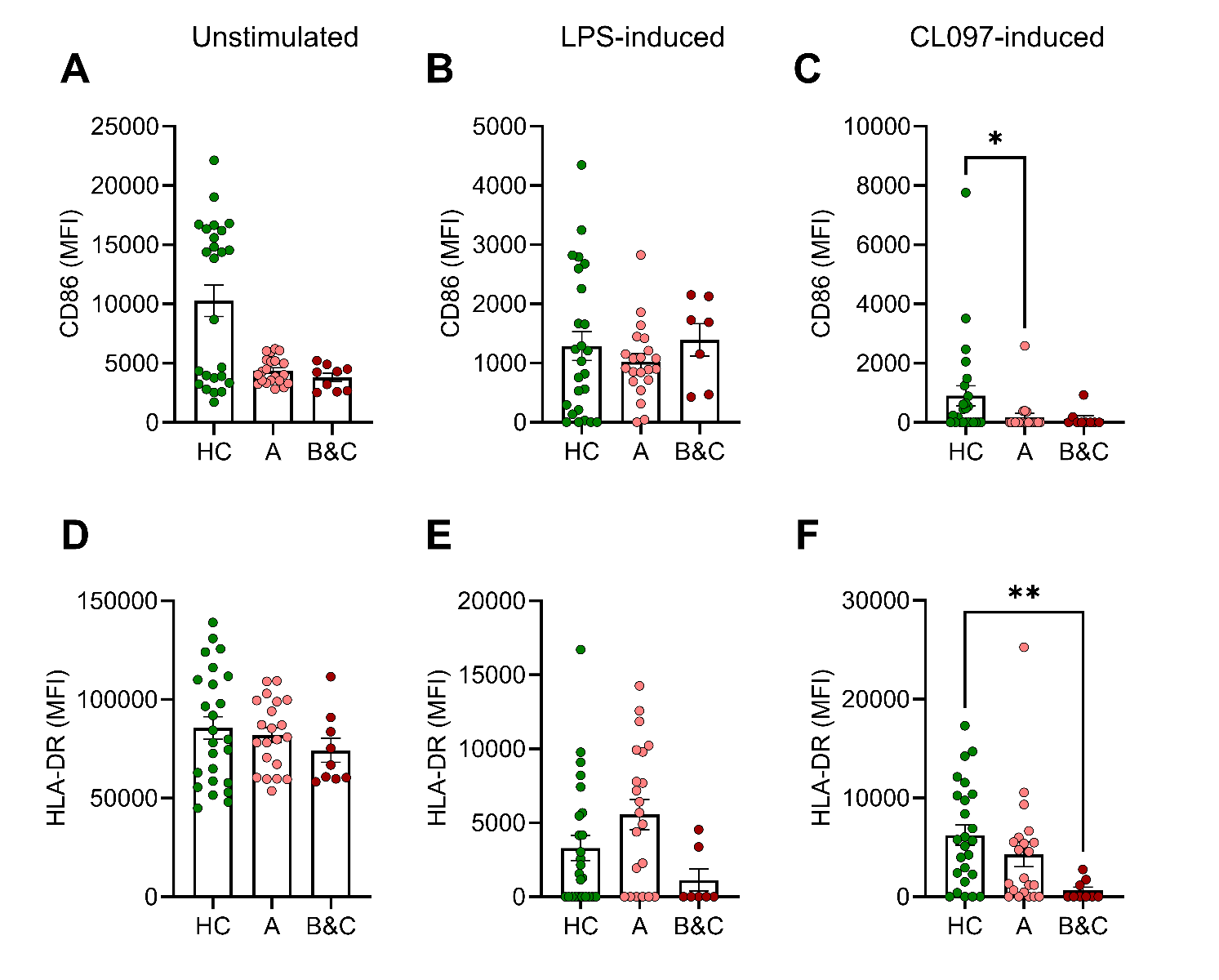


**Supplementary figure 2. Altered expression of CD86 and HLA-DR on conventional dendritic cells type-2 from cirrhotic patients.** (A, D) Basal, (B, E) LPS-induced and (C, F) CL097-induced expression of (A-C) CD86 or (D-F) HLA-DR on cDC2 from healthy controls (HC, N=25) and patients with cirrhosis (N=30) were determined by flow cytometry after five hours of culture. Cirrhotic patients are grouped by Child-Pugh class with patients in class A in one group (A, N=21), and class B and C in one group (B&C, N=9). Statistical analyses were performed using Kruskal-Wallis’ test with Dunn’s multiple comparisons test. *P<0.05, **P<0.01. MFI; median fluorescence intensity.


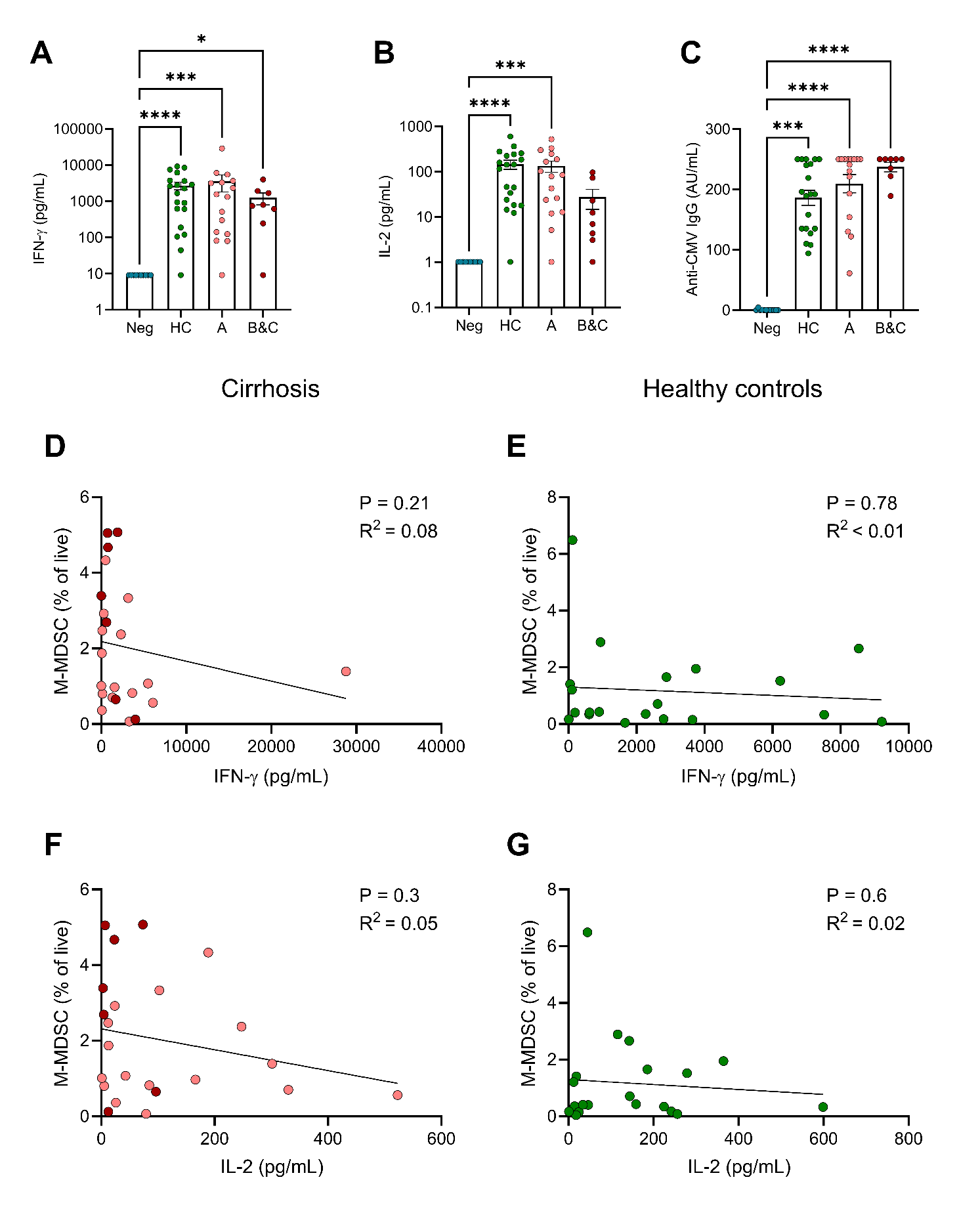
**Supplementary figure 3. Frequency of M-MDSC cells from cirrhotic patients and healthy controls do not correlate with CMV-induced T cell immune responses.** Cytomegalovirus (CMV) peptide-induced formation of (A) IFN-γ and (B) IL-2 in seronegative patients and controls (Neg, N=12) and seropositive healthy controls (HC, N=20) and patients with cirrhosis (N=24). Cirrhotic patients are grouped by Child-Pugh class with patients in class A in one group (A, N=16), and class B and C in one group (B&C, N=8). Associations between frequency of monocytic myeloid-derived suppressor cells (M-MDSC) and CMV peptide-induced (A-B) IFN-γ and (C-D) IL-2 in (A, C) cirrhotic patients (N=23) and (B, D) healthy controls (N=20). (A, C) Light red; Child-Pugh class A (N=16), dark red; Child-Pugh class B&C (N=7). Statistical analyses were performed using (A-B) Kruskal-Wallis’ test with Dunn’s multiple comparisons test or (C-F) Spearman’s rank correlation. *P<0.05, ***P<0.001, ****P<0.0001. MFI; median fluorescence intensity.
